# Interprofessional education: evaluation of a radiation therapy and medical physics student simulation workshop

**DOI:** 10.1002/jmrs.256

**Published:** 2018-01-23

**Authors:** Yobelli A. Jimenez, David I. Thwaites, Prabhjot Juneja, Sarah J. Lewis

**Affiliations:** ^1^ Faculty of Health Sciences The University of Sydney Lidcombe New South Wales Australia; ^2^ Institute of Medical Physics School of Physics University of Sydney Sydney Australia

**Keywords:** Interprofessional education, medical physics, radiation therapy, student education

## Abstract

**Introduction:**

Interprofessional education (IPE) involves two or more professions engaged in learning with, from and about each other. An initiative was undertaken to explore IPE for radiation therapy (RT) and medical physics (MP) students through a newly developed workshop based around simulated learning. The aims of this study were to explore RT and MP students’ perceptions of working as part of a collaborative team and of their own and the other group's professional roles. Student perceptions of the simulation education tool, the virtual environment for radiotherapy training (VERT) system, were also investigated.

**Methods:**

RT and MP students were invited to participate in a 4‐hour interprofessional workshop. Pre‐ and post‐workshop surveys were employed to collect demographic data, students’ perceptions of interdisciplinary education (interdisciplinary education perception scale (IEPS)) and workshop evaluation (bespoke questionnaire).

**Results:**

Fifteen students attended the workshop (RT, *n* = 8; MP, *n* = 7). Thirteen pre‐ and post‐questionnaires were returned (Pre‐questionnaire: RT, *n* = 6, response rate, 75%; MP, *n* = 7, response rate, 100%; post‐questionnaire: RT, *n* = 7, response rate, 87.5%; MP, *n* = 6, response rate 85.7%). For both student groups combined, IEPS scores ranged from 64 to 108 and 71 to 108 in the pre‐ and post‐questionnaires, respectively, with insignificant differences in the mean scores post‐intervention (*Z* = −1.305, *P* = 0.192). Satisfaction with VERT as a simulation tool was high for both student groups.

**Conclusions:**

The interprofessional student workshop served to promote interprofessional collaboration for RT and MP students. VERT was reported as an appropriate education tool for this purpose, enabling access to virtual clinical equipment common to both student groups. It is suggested that IPE continues to be offered and investigated in RT and MP students, in order to improve effective interprofessional strategies which may enrich future professional collaboration.

## Introduction

Interprofessional teams have been associated with improved health care delivery. This has been attributed to the joint intellectual effort of health professionals working together and exploiting skills and knowledge from each individual within the team.^1^ In radiation oncology, patient treatment requires a complex technical infrastructure and interprofessional human resources. Radiation therapy (RT) and medical physics (MP) professionals form part of the wider radiation oncology collaborative team, where practitioners partake in a cooperative process of profession‐specific activities, communication and decision making which influences accurate management, planning and delivery of RT.^2^ Evidence of attitudes and collaborative accomplishments between RT and MP professionals are limited in the literature,^3^, ^4^ with a general consensus that RT and MP staff should have an understanding of each other's roles, with well‐established working relationships.^5^, ^6^, ^7^, ^8^ A report from the World Health Organization,^1^ suggests that interprofessional collaboration can be enriched through interprofessional education (IPE).

IPE generally involves two or more professions engaged in learning with, from and about each other.^1^ It has been associated with enhanced motivation to collaborate and to cultivate interpersonal, group and organisational relations, having the ability to establish common values and knowledge bases and serve to reinforce competence.^9^, ^10^ In one of the few reports on IPE involving RT and MP students, Gillan et al^11^ reported on a team simulation event involving a group of 21 RT students, radiation oncology and MP trainees. Post‐intervention data indicated that participants highly valued interprofessional communication, clinical knowledge, clinical decision making, clinical skills and exposure to other trainees. In another study,^7^ RT and MP students were involved in non‐profession‐specific activities, such as clinical and educational conferences, with reported outcomes indicating increased knowledge and team communication, as well as less anxiety when communicating with radiation oncologists. Improved attitudes towards interprofessional teams and learning were also reported in an IPE programme involving medical, physiotherapy and RT students.^12^ In Australia, RT and MP students seldom collaborate in structured programmes during their higher education training, despite obvious synergies of content and professional duty of care.

An initiative was undertaken to explore IPE for RT and MP students, through a newly developed interprofessional student workshop. The workshop had three main objectives:

- for students to develop their own professional roles
- to enhance students’ understanding of their own and the other profession's role within the radiation oncology context.
- to characterise the importance of good communication for team work and patient care within radiation oncology.

Supported by positive experience using the virtual environment for radiotherapy training (VERT)(Vertual, Hull, United Kingdom) in Australia and overseas,^13^, ^14^, ^15^, ^16^ the workshop integrated VERT as a simulation education tool. To date, there are no published studies on the use of VERT in IPE for combined RT and MP student cohorts.

This article reports on the interprofessional student workshop evaluation. The primary aim of this study was to explore RT and MP students’ perceptions of their own profession and the other group's profession, and to investigate perceptions of RT and MP students working as part of a collaborative team. It was hypothesised that the workshop would improve students’ perceptions of interprofessional collaboration. A secondary aim was to investigate students’ experience with the VERT system. This information will contribute to further IPE initiatives, informing interprofessional developments within radiation oncology.

## Methods

### Ethical issues

Ethics approval for this study was granted by the Human Research Ethics Committees from The University of Sydney. Consent to participate in the study was given by return of questionnaires and all data collected was anonymous. The primary investigator was not academically associated with either student group.

### Study design

This study consisted of a ‐group, pre‐ and post‐intervention test design, using survey instruments for data collection.

### Workshop and study participants

The ‘*interprofessional student workshop’* was offered to RT and MP students who were enrolled at two different universities. RT students were enrolled in the second year of the undergraduate Bachelor of Medical Radiation Sciences (RT) stream programme at Charles Sturt University and MP students were enrolled in the first year of the Master of Medical Physics programme at The University of Sydney. These student groups were identified as being at near equivalent stages of theoretical knowledge and clinical experience in terms of the stage through their respective courses, most appropriate to the workshop's learning objectives. All eligible students (RT, *n* = 8; MP, *n* = 22) were invited to attend the *interprofessional student workshop* via email communication and/or direct communication by academic staff. The workshop was offered as an extra‐curricular activity. The invitation included a ‘Participant Information Statement’, outlining the workshop details and objectives. Information clearly stated that participation in the workshop did not oblige participation in the study and there would not be detriment to students who chose not to take part. The study was designed to include all RT and MP students who attended the workshop. There was no sampling or selection relevant to this study.

### Interprofessional student workshop

The workshop was four hours in duration and was intended for students at an early stage of their course. Decisions about organisation of the structure, content and learning methods were guided by general educational theory, IPE literature, learning objectives (previously stated) and ensuring that the workshop was attractive to both groups of students in terms of content and relevance.

The workshop took place at The University of Sydney's VERT suite (Lidcombe, Australia). VERT is a back‐projection simulation system, which displays an interactive RT treatment room, equipment (such as linear accelerator, treatment couch, calibration systems) and virtual RT patient (including anatomical volumes, CT images, treatment plans, etc.) on a large wall‐sized screen.^17^ The VERT system was used to support facilitator demonstrations and hands‐on and peer supported activities. The primary author had extensive experience as a VERT educator and led the workshop, with two other academic staff assisting the workshop activities. Table 1 outlines the workshop structure and content.

**Table 1:** Summary of ‘Interprofessional student workshop’ content and VERT integration

Workshop activity	Content	VERT (simulation)
Introduction	Introduction and workshop overview Introduction to VERT	VERT demonstration
Introduction to clinical equipment and RT concepts via simulation	RT treatment room Linear accelerator Treatment couch Isocentre MP equipment Radiation safety Quality assurance equipment and procedures	Hands on activities: Operate virtual linear accelerator and treatment couch. Apply knowledge of field sizes and isocentre Perform quality assurance procedure. Apply IGRT concepts.
Interprofessional issues and communication	Interprofessional discussion of clinical scenarios in radiation oncology, which address: Professional roles Collaborative practice Working relationships Communication	
RT patient and pathway.	RT patient: Pathway Planning and treatment	Hands on activities: Planning data and RT dose RT patient set up IGRT Quality assurance processes

VERT, virtual environment for radiotherapy training; RT, radiation therapy; MP, medical physics; IGRT, image‐guided radiation therapy.

### Data collection

The study period was from May, 2015 to June, 2015. Surveys were created in SurveyMonkey (Palo Alto, United States of America) and distributed through paper‐based surveys (pre‐survey) or online (post‐survey). This design was chosen because it allowed for cost‐effective, rapid data collection and was feasible for the intended study participants. Surveys were anonymous and participants were asked to provide a linking identification code in order to match pre‐ and post‐responses. The pre‐session survey was completed by participants prior to the workshop. Post‐session data were collected via an online survey which was available immediately after the completion of the workshop. Reminder emails were sent to MP students 1 and 2 weeks following the workshop. Consent to participate in the study was given by survey return. Surveys consisted of four sections:

- Demographic data
  This section asked students to indicate their gender (male, female, prefer not to say), age range (18–24, 25–34, 35–44, over 45, prefer not to say) and course enrolment (RT, MP). Students were also asked to indicate if they had any prior experience with the VERT system.
- Interdisciplinary Education Perception Scale (IEPS)
  The ‘interdisciplinary education perception scale’ (IEPS) was used to explore students’ perceptions of IPE.^18^ The IEPS consists of 18 items measured on a 6‐point Likert scale (1 = strongly disagree to 6 = strongly agree), with four subscales assessing ‘competence and autonomy’, ‘perceived need for cooperation’, ‘perception of actual cooperation’ and ‘understanding others’ value’. The total possible IEPS scores range from 18 to 108, with higher scores indicating better perception of interprofessional learning.
- Workshop evaluation
  A questionnaire was designed to explore students’ perception of the workshop and the VERT system. In the pre‐ and post‐questionnaires, students were asked to indicate how they perceived the workshop related to their university course (free‐text response). The post‐questionnaire asked students to indicate their level of agreement of 13 statements relating to the workshop (3, 2, 5 and 3 items for knowledge & understanding, content, VERT display and VERT system use respectively), using a 6‐point Likert scale (1 = strongly disagree to 6 = strongly agree). Higher scores indicate better agreement with posed statements.

### Data analysis

Descriptive statistics (frequency, mean and median) were calculated for demographic results (pre‐ and post‐questionnaire), workshop evaluation (13 items, post‐questionnaire only) and IEPS subscales (4 subscales, pre‐ and post‐questionnaire). In order to test for statistical significance, each participant was given an overall score on each of the four IEPS subscales for pre‐ and post‐workshop questionnaires and scores were combined for RT and MP student groups. A Wilcoxon signed‐rank test was used to compare pre‐ and post‐ survey means for each of the four IEPS subscales using IBM Statistical Package for the Social Sciences (IBM SPSS) for windows (Version 21). Statistical significance was set at 0.05 for all scales. Free‐text responses were independently reviewed by two of the authors [YJ, SL] and classified into themes. Final themes were reached by consensus.

## Results

Fifteen students (8 RT, 7 MP) attended the interprofessional student workshop. Participation in the workshop did not oblige participation in the study which involved completion of pre‐ and post‐session questionnaires. Whilst the pre‐ and post‐questionnaire included a coding system to match respondents, student non‐compliance with this section did not allow for this. In addressing missing data in the questionnaires, the reported results identify the number of respondents who completed each section.

### Demographic data

There were 13 completed pre‐session questionnaires returned from six RT and seven MP students (response rate for RT and MP students was 75 and 100% respectively). The same number of post‐session surveys were returned from seven RT and six MP students (response rate for RT and MP students was 87.5 and 85.7% respectively). Demographic data are described in Table 2. The majority of students had not previously seen the VERT system (*n* = 10), the remaining students had seen the VERT system (*n* = 2) or were unsure (*n* = 1). The two students who had seen the VERT system were enrolled in RT.

**Table 2:** Demographic data for pre‐ and post‐survey respondents

	Pre‐survey			Post‐survey		
	RT *N* (%)	MP *N* (%)	RT and MP *N* (%)	RT *N* (%)	MP *N* (%)	RT and MP *N* (%)
Total	6 (46.2%)	7 (53.8%)	13 (100%)	7 (53.8%)	6 (46.2%)	13 (100%)
Gender						
Male	2 (15.4%)	3 (23.1%)	5 (38.5%)	3 (23.1%)	3 (50%)	6 (46.2%)
Female	4 (30.7%)	4 (30.7%)	8 (61.5%)	4 (30.7%)	3 (50%)	7 (53.8%)
Age range						
18–24	5 (38.5%)	5 (38.5%)	10 (76.9%)	5 (38.5%)	5 (38.5%)	10 (77%)
25–34	1 (7.7%)	1 (7.7%)	2 (15.4%)	2 (15.4%)	1 (7.7%)	3 (23%)
35–45	–	1 (7.7%)	1 (7.7%)	–	–	–

RT, radiation therapy; MP, medical physics; N, number of students, (%) Percentage of students rounded to nearest one decimal place.

### Interprofessional education perception scale

A total of 11 out of 13 and 12 out of 13 returned questionnaires had complete IEPS sections, in the pre‐ and post‐questionnaire respectively. For RT and MP groups combined, the total IEPS scores ranged from 64 to 108 in the pre‐questionnaire and 71 to 108 in the post‐questionnaire. A Wilcoxon signed‐rank test on total IEPS scores showed that the ‘interprofessional student workshop’ did not elicit a statistically significant change in students’ perceptions of IPE (*Z* = −1.305, *P* = 0.192). There was also no statistically significant change in the four subscales (‘Competency and autonomy’: *Z* = −0.579, *P* = 0.563; ‘Perceived need for cooperation’: *Z* = −1.37, *P* = 0.891; ‘Perception of actual cooperation’: *Z* = 0.923, *P* = 0.356; ‘Understanding others’ value’: *Z* = −0.850, *P* = 0.395). The mean scores and standard deviation for total scores and subscales are listed in Table 3.

**Table 3:** Mean IEPS scores for RT and MP groups in the pre‐ and post‐intervention questionnaires

	Pre‐questionnaire			Post‐questionnaire		
	RT (*n* = 5) Mean (SD)	MP (*n* = 6) Mean (SD)	RT and MP (*n* = 11) Mean (SD)	RT (*n* = 7) Mean (SD)	MP (*n* = 5) Mean (SD)	RT and MP (*n* = 12) Mean (SD)
Total IEPS^a^	**93.6** (3.85)	86.5 (14.28)	89.7 (11.03)	**94.3** (6.55)	89.4 (13.16)	92.3 (9.63)
IEPS subscales^a^						
Competency and autonomy	**42.4** (2.88)	37.5 (7.15)	39.7 (6.00)	**42.4** (3.36)	39.2 (6.38)	41.1 (4.87)
Perceived need for cooperation	**11.8** (0.45)	10.5 (1.64)	11.1 (1.38)	**11.7** (0.49)	10.2 (1.48)	11.1 (1.24)
Perception of actual cooperation	**26.4** (2.07)	24.8 (3.54)	25.5 (2.94)	**26.6** (1.99)	25.8 (3.27)	26.3 (2.49)
Understanding others’ value	13.0 (1.41)	**13.7** (2.66)	13.4 (2.11)	13.7 (2.21)	**14.2** (2.49)	13.9 (2.23)

IEPS, interdisciplinary education perception scale; RT, radiation therapy; MP, medical physics; SD, standard deviation.

a Bold indicates higher mean (between RT and MP groups).

### Workshop evaluation

A total of 11 out of 13 students who completed the pre‐questionnaire (RT, *n* = 6; MP, *n* = 5) responded to the pre‐questionnaire section asking how well they perceived the workshop fitted with their university course. There were two main themes identified within students’ free‐text responses via the frequency of the themes mentioned. A common theme was students’ positive expectations of the potential value of the workshop, although this was expressed mainly from their own student‐practitioner viewpoint. Students gave examples of perceived benefits such as:

- *‘I think it is extremely important to be a part of this workshop. It will be great for a deeper understanding of the practical side of our professions’*. (RT, female)
- *‘It will be useful to build a better understanding of the role of medical physics in radiation therapy’* (MP, male)

A second theme involved students’ view of the perceived benefits of practicing skills in a virtual environment, as one student expressed:

- *‘…giving the students a chance to experience the workplace without taking away from patient care’* (RT, male)

In the post‐questionnaire, seven out of 13 students who completed the post‐questionnaire (RT, *n* = 5; MP, *n* = 2) responded to the question asking how well they considered the workshop fitted with their university course. Overall, students responded positively at the completion of the workshop, with a high regard for the VERT system. The first theme included students’ subjective feeling that the workshop supported understanding of their professional roles:

- *‘Training under the VERT workshop achieved a good understanding of the profession’* (RT, male)

The second theme identified included students’ ideals of learning as an interprofessional group:

- *‘…the course gave me a clear indication of how a linac works and how to operate it, as well as introduced me to the role of a medical physicist in a radiation oncology clinic. I also enjoyed learning about the role of a radiation therapist and getting to meet and talk to some radiation therapists’*. (MP, male)
- *‘The VERT system itself was great, but combining the MP and RT was semi pointless. I thought the RT students were more advanced and had to wait for the MP students to catch up. However, playing with the VERT system was great fun’*. (RT, female)

In the post‐questionnaire, a total of seven RT students completed questions about VERT display and use (100% response rate). One RT student did not complete the knowledge and understanding section, hence only six RT students completed this section. Three MP students completed this section of the questionnaire (50% response rate). MP students’ scores had higher means for questions addressing the workshop's effect on knowledge and understanding. The workshop content had good response to relevance in both student groups and was not considered difficult to understand. All the VERT display features identified were highly rated, by both student groups and there was a high regard for using VERT simulation as the central learning tool for the workshop. Statistics are summarised in Table 4.

**Table 4:** Score statistics for interprofessional student workshop evaluation^a^

	RT (*n* = 7)		MP (*n* = 3)		RT and MP (*n* = 10)	
	Mean (SD)	Median (Range)	Mean (SD)	Median (Range)	Mean (SD)	Median (Range)
Knowledge and understanding^b^						
The workshop increased my knowledge about the roles and duties of RT and MP professionals	3.2 (1.2)	3 (2–5)	5.0 (1.0)	5 (4–6)	3.8 (1.4)	4 (2–6)
The workshop changed my understanding of how RT and MP professionals work together	3.5 (1.2)	4 (2–5)	4.3 (1.5)	4 (3–6)	3.8 (1.3)	4 (2–6)
RT and MP professionals work as a part of a collaborative team	5 (0.9)	5 (4–6)	5.3 (1.2)	6 (4–6)	5.1 (0.93)	5 (4–6)
Workshop content						
The content covered in the workshop was difficult to understand	2 (1.3)	1.5 (1–4)	3 (0.0)	3 (3–3)	2.3 (1.1)	3 (1–4)
The content covered in the workshop was relevant to me	4 (1.3)	3.5 (3–6)	5 (1.0)	5 (4–6)	4.3 (1.2)	4 (3–6)
Display on VERT (useful for understanding theoretical concepts)						
External contour	4.7 (1.5)	5 (2–6)	4.3 (0.6)	4 (4–5)	4.6 (1.3)	4.5 (2–6)
Internal anatomy	4.9 (1.5)	5 (2–6)	4.3 (0.6)	4 (4–5)	4.7 (1.3)	5 (2–6)
Radiation fields	4.6 (1.6)	5 (2–6)	5 (1)	4 (4–6)	4.7 (1.4)	5 (2–6)
CT images	4.6 (1.5)	5 (2–6)	4.3 (0.6)	4 (4–5)	4.5 (1.5)	5 (2–6)
Treatment machine	4.4 (1.5)	5 (2–6)	4.7 (0.6)	5 (4–5)	4.5 (1.3)	5 (2–6)
VERT system use						
I feel motivated and enthused as a result of using VERT	4.7 (1.0)	5 (3–6)	4.7 (1.2)	4 (4–6)	4.7 (0.9)	5 (3–6)
I enjoyed using VERT	5.3 (1.0)	6 (3–6)	4.3 (1.5)	4 (3–6)	5 (1.2)	5.5 (3–6)
I experienced difficulties using VERT	2.6 (1.0)	3 (1–4)	3 (0.0)	3 (3–3)	2.7 (0.8)	3 (1–4)

RT, radiation therapy; MP, medical physics.

a Higher score indicates higher level of agreement with the statement.

b RT group, *n* = 6 for this section (missing data omitted).

## Discussion

To our knowledge, this study was the first to utilise VERT in an interprofessional workshop setting for RT and MP students. Outcomes of students’ perceptions of IPE indicated positive changes post‐intervention, although our results cannot confirm that these changes were due to the educational intervention. IEPS mean scores increased from 93.6 to 94.3 in the RT student group and from 86.5 to 89.4 in the MP student group. Differences between RT and MP scores can be attributed to students’ own perceptions, as well as students being from different universities and study programmes, as researchers have found these factors influence attitudes to IPE.^19^, ^20^ IEPS mean scores for both student groups combined increased from 89.7 to 92.3 (*Z* = −1.305, *P* = 0.192). This may suggest the interprofessional student workshop was able to positively influence students’ perceptions of IPE, although this cannot be ascertained and is not supported by statistical significance. These scores are higher than previously reported IEPS scores for undergraduate medical, nursing and surgical technology students.^19^

For individual student groups, mean scores for three out of the four subscales (‘competency and autonomy’, ‘perception of actual cooperation’ and ‘understanding others’ value’) increased or remained the same in the pre‐ and post‐questionnaires for both student groups. Mean scores for ‘perceived need for cooperation’ decreased for both groups (*P* > 0.05). The reason for this is uncertain and difficult to justify for this small student sample.

When comparing IEPS subscale scores for RT and MP student groups, it was found that RT students had higher mean scores compared to MP students for three out of the four IEPS subscales in the pre‐ and post‐questionnaires. These were, ‘competency and autonomy’, ‘perceived need for cooperation’ and ‘perception of actual cooperation’. In previous reports, health professional student groups with higher IEPS scores had more years of study,^19^ which contributed to increased experience and interactions with other professional groups. Whilst both RT and MP student groups in our study were at an early stage of their RT‐ and MP‐specific university programmes, RT students were in their second year of study, and had prior health‐related interprofessional experience in their first undergraduate year of study and also had had more exposure to real‐world clinical environments and linear accelerator facilities at that stage. For ‘understanding others’ value’ subscale, MP students had a higher mean score in both the pre‐ and post‐questionnaire compared to RT students. In both the pre‐ and post‐questionnaires, the standard deviation for each of the IEPS subscales was higher for MP students, indicating wider variability in responses for this group. This may be due to MP students’ only recent exposure to RT and understanding of their role within radiation oncology.

The student workshop evaluation demonstrated that the interprofessional experiences provided to students maintained or increased students awareness of RT and MP professionals being part of a collaborative team. Furthermore, students indicated that the workshop had increased their knowledge about the roles and duties of RT and MP professionals. Interestingly, MP group had higher scores, indicating that perhaps their knowledge of relative RT and MP roles was lower prior to the IPE intervention. This was reinforced by RT student comments, which stated that the workshop may be more appropriate for more advanced MP students and should be offered at an earlier stage of the RT programme. Additionally, it was identified that RT students’ expectations of the workshop may have been skewed away from the IPE context, instead towards utilisation of the VERT system.

From a facilitators’ perspective, the VERT system was found to be an integral asset of the interprofessional VERT workshop. VERT provided access to virtual clinical equipment common to both student groups, which replicated a range of clinical and theoretical concepts, whilst allowing repetitive activities to be undertaken in a relatively short time frame. From the questionnaire results, there was a positive anticipation (prior to workshop) and experience (following workshop) of seeing and operating the VERT system, consistent with student experience of VERT in other studies.^13^, ^15^

### Limitations

The sample size for this study was small, therefore, this article relies on trends in survey response rather than statistically based evidence. In addition, some questions had very low response rate which likely compromises the reliability of the results from a statistical analytical viewpoint. Whilst this reduces the ability to generalise results to a greater population, the data collected support the exploratory nature of the study as well as future research into IPE for RT and MP students. Another limitation lies within the reliability of the IEPS scale for RT and MP student groups, although it has been used other health profession students.^19^, ^20^, ^21^, ^22^

It was not possible to include the interprofessional student workshop into RT or MP student curriculum. The interprofessional student workshop was therefore offered as an extra‐curricular activity, which can account for the low attendance rate by the MP group (31.8% of total enrolment). Nonetheless, this resulted in RT and MP students being almost equally represented.

### Future work

In the future, interprofessional simulation workshops could be integrated into university curriculums in order to improve participation rates, and incorporate other radiation oncology professional groups, such as radiation oncology registrars and nurses. It is currently unclear how MP and RT IPE at university level could influence interprofessional collaboration at a student level and, later, at practitioner level. Longitudinal studies could be undertaken, which specifically evaluate impacts on translation into practice.

## Conclusion

In conclusion, results from the workshop evaluation are encouraging and serve to promote educational activities where RT and MP students can learn with, from and about each other. Students’ interaction with virtual clinical equipment was positive, and VERT as a simulation education tool proved to be an appropriate and enjoyable tool. It is suggested that IPE continues to be evaluated in RT and MP students, in order to ascertain if these experiences can translate to improved professional collaboration, with positive effects to RT patient care.

## Conflict of Interest

The authors declare that there is no conflict of interest regarding the publication of this paper.

## Informed consent

Informed consent was obtained from all individual participants included in the study.
